# Exploring the Relationship of Anxiety and Depressive Symptoms and Impulsiveness with the Quality of Life of Older Patients with Cardiovascular Disease: A Cross-Sectional Study

**DOI:** 10.3390/ijerph21050646

**Published:** 2024-05-19

**Authors:** Giada Pietrabissa, Gloria Marchesi, Luca Alessandro Gondoni, Gianluca Castelnuovo

**Affiliations:** 1 Department of Psychology, Catholic University of Milan, 20123 Milan, Italy; gloria.marchesi03@icatt.it (G.M.); gianluca.castelnuovo@unicatt.it (G.C.); 2 Clinical Psychology Research Laboratory, IRCCS Istituto Auxologico Italiano, Ospedale San Giuseppe, 28824 Oggebbio, Italy; 3 Division of Cardiological Rehabilitation, IRCCS Istituto Auxologico Italiano, Ospedale San Giuseppe, 28824 Oggebbio, Italy; gondoni@auxologico.it

**Keywords:** anxiety, cardiovascular disease, depression, elderly, impulsiveness, quality of life

## Abstract

Background: This study aimed to evaluate the relationship of selected clinical (i.e., body mass index, BMI) and psychological factors (i.e., anxiety, depression, and impulsiveness) with the quality of life (QoL) of elderly patients with cardiovascular disease (CVD) in a single clinical center in Italy. Methods: A total of 238 patients of older age (≥65 years) with CVD who voluntarily attended a single clinical center for weight loss and cardiac rehabilitation were sequentially recruited and tested upon admission to the hospital based on pre-established inclusion criteria. Results: The findings indicated that anxiety and depressive symptoms were moderately associated with lower QoL. Additionally, there were noteworthy but minor negative connections between impulsivity and QoL. Furthermore, BMI was inversely associated with the perceived QoL of the participants, and when incorporated into the regression analysis, BMI alone significantly accounted for 11.8% of the variability in QoL. This percentage increased to 18.4% with the inclusion of impulsiveness in the model and further to 34.3% with the addition of anxiety and depressive symptoms. However, after introducing anxiety and depression, the association between impulsivity and QoL ceased to be statistically significant. Conclusions: Integrating the routine assessment and treatment of psychological factors into the care of older patients with CVD is important for optimizing their overall health outcomes and improving their QoL.

## 1. Introduction

Cardiovascular diseases (CVDs) are a group of disorders of the heart and blood vessels [1]. Despite great advances in medical science and technology in its prevention, diagnosis, and treatment, CVD remains a significant public health concern globally, with the majority of deaths attributed to CVD-related complications [2].

In Italy, the prevalence of CVD is nearly double the global average [2]. From a public health perspective, this results in a substantial economic burden of the disease, accounting for one in every seven hospital admissions and resulting in expenditures exceeding EUR 3 billion on medications [3].

Although aging is not directly responsible for CVD, research indicates that the prevalence of CVD increases with population aging [4,5,6,7,8,9,10,11,12,13]. Various factors contribute to this association, including natural age-related alterations within the cardiovascular system and other bodily systems over time. In addition, geriatric syndromes, such as neurological decline, functional limitations, or comorbidities, can delay cardiovascular recovery [14,15].

Italy is one of the nations with the highest life expectancy in Europe and globally [10].

Therefore, the global burden of CVD, coupled with the rapid growth of the aging population, places increased strain on healthcare systems and society to identify potential determinants of adverse outcomes [16].

Quality of life (QoL) reflects an individual’s subjective perception of their health status, overall functioning, and well-being, including the physical, mental, emotional, and social functioning domains [17]. It is considered a significant benchmark for modern healthcare outcomes in individuals with CVD [18,19] and also plays an increasing role in understanding healthy aging [9,20,21].

A German study [22] revealed that QoL among patients with CVD was generally on par with that of the general German population. However, a negative correlation was found between QoL and age, indicating that QoL declines more rapidly in older individuals with CVD compared to the community sample. Various sociodemographic, physiological, and psychological factors can influence the perceived QoL of seniors with CVD [21,23,24]. Among them, the coexistence of other comorbidities, such as obesity or overweight issues, along with CVD was significantly associated with a lower QoL [25].

The obesity epidemic is a critical global public health concern and stands as a significant contributor to the worldwide burden of chronic illness and disability, with serious social and psychological implications. Patients with obesity are a common profile upon admission to cardiac rehabilitation (CR) programs [26], and research reveals that a higher BMI in elderly people might decrease life expectancy, increase metabolic disturbances such as elevated blood pressure and dyslipidemia, and reduce cardiovascular health [27,28,29,30].

Additionally, symptoms of anxiety and depression can have a profound impact on the subjective state of health of elderly people with CVD [31] and are associated with an increased risk of developing obesity during the transition to older adulthood [32,33,34,35,36].

Symptoms of anxiety and depression are four to five times more prevalent in patients with CVD than in the general population [37,38,39,40], and there is compelling evidence for their association with the QoL of individuals, even after controlling for biological aspects [41,42]. Untreated psychological distress can lead to nonadherence to medical regimens [43,44], unhealthy lifestyle behaviors, and an increased risk of adverse cardiovascular events [36,45]. Furthermore, anxiety and depressive symptoms can complicate CVD management by amplifying pain perception, altering sleep, and exacerbating fatigue, all of which can further hinder recovery and rehabilitation efforts [46,47]. This holds particular significance for the elderly, as their QoL is notably influenced by their functional capabilities and health status to a greater extent compared to younger age cohorts [9,21]. Recognizing and addressing these mental health concerns is, therefore, essential for optimizing cardiovascular outcomes and improving the QoL of elderly individuals with CVD [48,49,50].

Moreover, research exists on the association between the type A behavior pattern (TABP), characterized by an intense drive and competitive behavior, and CVD [51,52]. However, the specific role of impulsivity, a characteristic of the TABP, in predicting the QoL of patients with CVD remains understudied [53]. Impulsivity is a personality trait defined by the tendency to act on immediate urges or impulses without considering potential consequences [54].

Research has indicated a detrimental impact of impulsivity on the cardiovascular system through an unfavorable lifestyle and biological actions that include endothelial damage and exaggerated reactivity to environmental stressors [23,55].

Increased impulsivity among older people was also correlated with a reduced probability of exercising regularly and a greater tendency to engage in CVD-risk behaviors [53]. Moreover, a recent study revealed that impulsivity was longitudinally associated with decreased adherence to healthy eating habits over three years in subjects with CVD and comorbid overweight or obesity [56].

However, the exact nature of the interplay between these factors is complex, and further research is needed to understand the association of impulsivity with QoL.

This is particularly important among the older cardiac population as, to our knowledge, little is known about the collective impact of BMI and relevant psychological factors on the QoL of the Italian geriatric population with CVD.

Therefore, exploring the relationships between BMI, anxiety and depressive symptoms, impulsiveness, and QoL among older patients diagnosed with CVD can improve the understanding of the nuanced factors that shape their QoL, thus informing targeted interventions and holistic care approaches [57,58].

This cross-sectional study aims, for the first time, to elucidate the relationship between selected clinical (body mass index) and psychological factors (anxiety, depression, and impulsiveness) and the QoL of a sample of elderly Italian patients with CVD and comorbid obesity.

Consistent with the growing body of literature, we hypothesized that elevated BMI, depressive and anxiety symptoms, and impulsiveness correlate with decreased QoL in the sample.

## 2. Materials and Methods

The study used a cross-sectional design.

All procedures performed in this study were in accordance with the ethical standards of the institutional and/or national research committee and with the 1964 Helsinki Declaration and its later amendments or comparable ethical standards. Ethical approval was obtained from the Ethics Committee of the IRCCS Istituto Auxologico Italiano (protocol ID: 03C202_2002).

### 2.1. Study Participants

A total of 238 patients with CVD who voluntarily attended a single clinical center (San Giuseppe Hospital, IRCCS Istituto Auxologico Italiano) for weight loss and cardiac rehabilitation (duration 25 ± 3 days) from April 2018 to June 2019 were sequentially recruited upon admission to the hospital.

Patients were included in the study if (1) they were 65 years or older; (2) had Italian nationality; (3) had CVD in their medical history, including previous myocardial infarction, coronary angioplasty, or coronary artery bypass; heart failure with reduced or preserved ejection fraction; and valvular heart disease. Exclusion criteria were cognitive or visual impairment or psychiatric illness. All patients were in a medically stable state, and people who had experienced acute events in the past month were not included in the study.

### 2.2. Procedure

On admission to the hospital, the inpatients were asked to complete a validated Italian translation of a questionnaire consisting of selected self-report measures. The questionnaires were administered in group settings for convenience by a clinical psychologist independent of the study. The purpose of the investigation was explained, and all patients signed a written and informed consent form to participate in the study.

Demographic information, such as age, sex, marital status, employment status, and education, and clinical data, including smoking status, weight, and height (used to derive the participants’ BMI, calculated as body weight in kilograms divided by the square of height in meters), were also collected and retrieved from a secure electronic database of medical records.

The selection of participants from a clinic specializing in obesity and cardiovascular diseases suggests that those included in the study were actively seeking medical attention for these particular health issues. Therefore, it is crucial to include BMI as an independent variable to ensure the study’s integrity and thoroughness, especially considering the specific population being examined. Moreover, we focused on gathering data related to the aforementioned variables due to their ease of assessment and lower associated costs compared to factors like dyslipidemia and hypertension, which may necessitate additional tests. These additional tests could pose challenges, particularly for elderly individuals, and might hinder participants’ willingness to consent to participation.

### 2.3. Measures

The Quality of Life Index-cardiac version (QLI) [59] comprises two sections, each with 35 questions scored on a 6-point Likert. The first section reflects the satisfaction of people with various aspects of life (ranging from 1 = ‘very dissatisfied’ to 6 = ‘very satisfied’), while the second section measures the importance of the same aspects for the person (ranging from 1 = ‘very unimportant’ to 6 = ‘very important’). The QLI produces five scores ranging from 0 to 30: a total quality-of-life score and four main dimensions of QoL: (1) health and functioning, (2) socio-economic aspects, (3) psychological and spiritual status, and (4) family and relationships, and (5) the overall QoL. Higher scores indicate a better QoL. For the total scale, internal consistency reliability is supported by Cronbach’s alphas ranging from 0.73 to 0.99 in 48 studies, while evidence of temporal reliability was provided by test–retest correlations of 0.87 with a two-week interval and 0.81 with a one-month interval [59] and by a correlation of 0.78 with a three- to four-week interval [60]. In addition, support for the validity of the scale was provided by the fact that the items were based on both an extensive review of issues related to quality of life and patient reports regarding the quality of their lives by Ferrans and Powers (1985) [59] and an acceptably high rating using the content validity index [61]. The construct validity was determined by factor analysis, which revealed that the four-factor solution explained 91% of the total variance in the factor analysis, which revealed four dimensions underlying the QLI: health and functioning, social and economic, psychological/spiritual, and family [62,63]. Cronbach’s alpha of the QLI_total scale was 0.72 in the present population.

The Hospital Anxiety and Depression Scale (HADS) [64,65] consists of 14 items scored on a 4-point Likert scale (ranging from 0 to 3) to assess levels of anxiety (HADS_Anxiety) and depression (HADS_Depression) in individuals (7 items for each subscale). The total score is derived by summing the responses to all 14 items, while the subscale scores are calculated by summing the respective 7 items, yielding scores that range from 0 to 21 for each subscale. Subscale scores of 8 or higher indicate clinically elevated symptoms and can be further categorized as mild (score 8–10), moderate (score 11–14), or severe (score 15–21).

The bi-factorial structure of the HADS was confirmed in its original Italian validation article, which also showed that it was invariant between age and gender groups and had Cronbach’s alpha reliability coefficients of 0.86 and 0.80 for anxiety and depression, respectively [65]. In the present study, the internal consistency (Cronbach’s alpha) of the HADS was 0.81 for the anxiety dimension and 0.78 for the depression subscale.

The Barratt Impulsiveness Scale (BIS-11) [66,67] comprises 30 items scored on a 4-point Likert scale (ranging from 1 = rarely/never to 4 = almost always/always) designed to assess personality impulsivity tendencies. It is the most widely used single measure of impulsivity. The BIS-11 not only provides a total score (BIS_Total) but also delves deeper into the nuances of impulsiveness by offering six first-order factors, namely, attention, motor, self-control, cognitive complexity, perseverance, and cognitive instability impulsiveness. Additionally, it identifies three second-order factors, namely, attentional (BIS_Attentional impulsiveness), motor (BIS_Motor impulsiveness), and non-planning impulsiveness (BIS_No-planning impulsiveness), providing a comprehensive understanding of various facets within the personality and behavioral construct of impulsiveness. Total scores can range from 30 to 120, with higher scores reflecting higher levels of impulsiveness.

In the Italian BIS-11 validation article, Cronbach’s alpha for internal consistency was 0.79, and the reliability of the 2-month test–retest was 0.89 [67]. Similarly, a subsequent systematic review of the psychometric properties of the questionnaire found Cronbach’s alphas to range from 0.69 to 0.83 in 16 studies [68]. The instrument also demonstrated a moderate to large magnitude of reliability of the retest and good criterion-related validity in differentiating between clinical and non-clinical samples. Lastly, the correlations observed between dimensions ranged from 0.06 to 0.59, suggesting that BIS-11 factors tend to be associated but independent [68]. In the present sample, Cronbach’s alpha coefficient was 0.72, suggesting satisfactory scale homogeneity.

### 2.4. Statistical Analysis

Data were analyzed using the Statistical Package for the Social Sciences (SPSS) software (SPSS, version 20.0; SPSS, Inc., Chicago, IL, USA).

The demographic and clinical characteristics of the participants were calculated as frequencies and percentages for categorical variables and means and standard deviations for numerical variables.

When checking for normality, no significant violations were found, and there were no multivariate outliers detected. Therefore, Pearson’s *r* was used to test the associations between continuous variables (that is, BMI, HADS_Anxiety, HADS_Depression, BIS_Total, BIS_Attentional impulsiveness, BIS_Motor impulsiveness, and BIS_No-planning impulsiveness) and QLI scores (that is, QLI_Total, QLI_Health and functioning, QLI_Social and economic aspects, QLI_Psychological and spiritual status, and QLI_Family and relationships). The following hierarchical multiple linear regression was conducted using three models: (1) Model 1 predicted the QLI_total from BMI; (2) Model 2 added the BIS_Total score; and (3) Model 3 added HADS_Anxiety and HADS_Depression. The *p*-value was considered significant at <0.05 and highly significant at ≤0.01.

### 2.5. Sample Size Calculation

The sample size was estimated using N > 50 + 8 m (where m refers to the number of predictors in the model) [69]. Taking into account four independent variables (that is, BMI, BIS_Total, HADS_Anxiety, and HADS_Depression), 83 cases were needed [70,71].

## 3. Results

### 3.1. Sociodemographic and Clinical Characteristics of the Sample

The sample consisted of 168 men (70.6%) and 70 women (29.4%), giving a total of 239 respondents.

The ages ranged from 65 to 86 years, with a mean of 71.56 (SD = 5.05), while the BMI ranged from 18,4 to 54,5 kg/m^2^. In particular, 168 respondents (70.6%) had a BMI ≥ 30: 44 subjects (18.5%) had class I obesity, 70 individuals (29.4%) had class II obesity, and 52 patients (21.8%) had class III obesity. Furthermore, more than half of the respondents were married (n = 153; 64.3%) and retired (n = 209; 87.8%). Regarding their education, 89 people (37.4%) had a high school diploma, 76 people (31.9%) had a middle school diploma, and 54 (22.7%) and 19 (8.0%) respondents had an elementary school diploma and college degrees, respectively. Also, only 11% of the sample declared themselves as active smokers.

According to the HADS criteria, approximately one-third of respondents exhibited mild to severe symptoms of anxiety, while one in four patients displayed mild to moderate symptoms of depression. Descriptive statistics are presented in Table 1 below.

### 3.2. Correlation of Anxiety and Depressive Symptoms and Impulsivity with Quality of Life

Significant moderate negative correlations were found between symptoms of depression and all QoL dimensions. QLI_Total: r = −0.565, *p* < 0.001; QLI_health and functioning: r = −0.538, *p* < 0.001; QLI_social and economic: r = −0.414, *p* < 0.001; QLI_ psychological and spiritual: r = −0.559, *p* < 0.001; QLI_family and relationships: r = −0.412, *p* < 0.001.

Similarly, anxiety symptoms were moderately associated with QoL, except in the family and relationships domain, with which a small correlation was observed (r = −0.279, *p* < 0.001). Statistically, albeit small, significant negative correlations were also observed between the total impulsiveness score and the non-planning impulsiveness dimension and the perceived global QoL of elderly respondents (r = −0.163, *p* < 0.005; r = −0.167, *p* < 0.005, respectively), as well as with the health and functioning (r = −0.159, *p* < 0.005; r = −0.165, *p* < 0.005), the social and economic aspects (r = −0.198, *p* < 0.001; r = −0.183, *p* < 0.001), and the psychological and spiritual state (r = −0.166, *p* < 0.0055; r = −0.178, *p* < 0.001) domains of QoL, as measured by the QLI. In contrast, no statistically significant associations were found between any dimensions of impulsiveness and the QoL family and relationships domain. Specifically, the results showed no significant correlation with either the QLI_total score or any of its specific subscales. Instead, only a small significant negative correlation was observed between attentional impulsiveness and the social and economic aspects of QoL (r = −0.138, *p* < 0.005). Lastly, significant, though small, negative correlations were found between BMI and all dimensions of QoL. QLI_Total: r = −0.344, *p* < 0.001; QLI_health and functioning: r = −0.374, *p* < 0.001; QLI_social and economic: r = −0.295, *p* < 0.001; QLI_ psychological and spiritual: r = −0.257, *p* < 0.001; QLI_family and relationships: r = −0.235, *p* < 0.001. Please see Table 2 below.

### 3.3. Predictors for Quality of Life of Elderly Patients with CVD

A hierarchical linear regression analysis was performed to evaluate the prediction of QoL by BMI, impulsiveness, anxiety, and depression (Table 3).

In Model 1, BMI represented a significant amount of variance in the QoL of elderly patients with CVD [F(1,231) = 30.9, *p* = 0.000, R^2^ = 0.114]. In Model 2, the predictor variable impulsiveness was added to the analysis. The results revealed a significant increase in variance that was responsible for QoL [F(2,230) = 18.441, *p* = 0.021, R^2^ = 0.131]. In Model 3, the predictor variables anxiety and depression were further added to the model. This led to another significant increase in variance that accounted for QoL [F(4,228) = 34.351, *p* = 0.000, R^2^ = 0.365]. In general, the variables explained 37.6% of the variance in the QoL of elderly patients with CVD.

## 4. Discussion

To our knowledge, this was the first study in which the relationship between the clinical (BMI) and psychological parameters and QoL of older Italians with CVD was examined in a comprehensible way.

Specifically, although there is evidence for an association between BMI and symptoms of anxiety and depression and QoL in patients with CVD [72,73,74,75], especially those of older age [76], the construct of impulsivity has been less investigated in association with CVD in the elderly [77,78], and no study so far has investigated the relationship between all of these variables taken together and the QoL of elderly people with CVD in Italy.

The results support previous research suggesting that symptoms of depression and anxiety are negatively associated with QoL and other relevant clinical outcomes in patients with CVD [79,80,81,82,83,84,85,86,87,88,89,90].

Moreover, significant, albeit small, negative associations were found between impulsiveness and QoL. More detailed correlations were found between the total impulsiveness score and the non-planning impulsiveness domain and all QoL dimensions, excluding that of family and relationships. This implies that higher levels of impulsiveness, particularly in the non-planning domain, are associated with a lower QoL in multiple areas. This finding could have significant implications for understanding how impulsiveness impacts different aspects of individuals’ lives, highlighting potential areas for intervention or support. Additionally, the exclusion of the family and relationships dimension from the correlations raises questions about potential factors or dynamics unique to these areas that may influence the relationship between impulsiveness and QoL in elderly patients with CVD. Further discussion and analysis could explore why these dimensions show different patterns of correlation and what implications this may have for understanding and addressing impulsiveness and its impact on QoL.

Still, these results should be interpreted considering that the large majority of the sample had obesity as a comorbidity, and, as shown in previous studies [55], both motor and attentional impulsiveness are hard to detect in overweight or obese patients.

Therefore, the relationship between impulsiveness and weight status may vary depending on the specific impulsiveness domain. This raises questions about the implications for behavioral patterns and health outcomes. Lower levels of motor and attentional impulsiveness in individuals with overweight or obesity could, indeed, potentially mitigate certain health risks associated with impulsive behaviors, such as overeating or substance abuse. However, the comparable levels of non-planning impulsiveness suggest that certain cognitive or decision-making processes may not be affected by weight status to the same extent.

The findings of this study also suggest that BMI negatively correlated with the perceived QoL of the sample, although this association was less strong than those that QoL was shown to have with anxiety and depressive symptoms. Accordingly, evidence exists for the negative impact of a higher BMI on the QoL of individuals, and obesity was associated with the causal promotion of CVD in different studies [23,55,91]. When entered into the regression model, BMI alone significantly predicted 11.8% of the variance in QoL, which increased to 18.4% when impulsiveness was added to the model. Lastly, the model comprising the variable mentioned above plus symptoms of anxiety and depression was able to account for 34.3% of the total variance in the QoL of elderly people with CVD. However, by adding anxiety and depressive symptoms, the association between impulsiveness and QoL was no longer statistically significant. A possible explanation for this outcome is that impulsivity might be totally mediated by depression. This is important since all of these emotional variables should be carefully considered and evaluated to provide adequate support to elderly patients with CVD.

A notable gap in the existing literature is the lack of studies that directly examine the relationship between impulsivity and QoL within a cohort of elderly patients diagnosed with CVD, both in Italy and globally. Consequently, the significance of this study lies in its investigation of these unexplored domains, offering a novel perspective on how symptoms of depression and anxiety and impulsivity correlate with the QoL of this specific sample. Consequently, this research offers valuable insights that could improve our comprehension of the intricate dynamics influencing the QoL of patients and drive future research questions.

### Limitations and Strengths of the Study

This study presents some limitations. First, its cross-sectional nature does not allow one to draw a cause-and-effect relationship between variables. Additionally, psychological measures were collected at one time, and this could be a momentary episode. Second, the data were obtained from a single center and, therefore, cannot be generalized to all older people with CVD in Italy. Third, participants may present comorbidities that have an impact on their perceived QoL. Different diseases might indeed have impacted their QoL differently, even with different pathways. Additionally, the indiscriminate inclusion of all cardiac conditions may have prevented the achievement of standardized measurement results. Also, all data were self-reported and, therefore, may be biased. Still, the use of a specific outcome measure for cardiac patients can represent a strength of this study, as it allows for a more accurate assessment of their QoL, providing clinicians with valuable information to improve patient-centered care. Moreover, including impulsivity among the psychological determinants of patients’ QoL not only enriches our understanding of the psychological characteristics of this specific population of patients and of how these factors interact with the subjective well-being of individuals with CVD older than 65 years but also informs targeted interventions to improve the effectiveness of treatment approaches aimed at improving QoL outcomes. Overall, the results should be interpreted considering these concerns, and more clinical research is needed, including longitudinal investigations, studies that test mediation models, and/or thematic analyses, to better explain and understand the associations between these parameters.

## 5. Conclusions

This contribution adds to the body of research indicating that older individuals with CVD experience a decreased QoL when presenting symptoms of anxiety and depression, impulsivity, and a higher BMI. This further highlights the importance of screening for the presence of emotional problems to offer specific support and interventions.

By implementing targeted support strategies, such as therapy, counseling, or psychoeducation programs, individuals can receive the resources and guidance necessary to effectively navigate and manage their psychological and health-related challenges.

## Figures and Tables

**Table 1:** Sample descriptive statistics.

	Descriptive Statistics	
Age (M, SD)	71.56	5.05
Sex (n, %)		
Male	168	70.6%
Female	70	29.4%
Civil Status (n, %)		
Single	18	7.6%
Married	153	64.3%
Separated/divorced	32	13.4%
Widower	35	14.7%
Education (n, %)		
Elementary school diploma	54	22.7%
Middle school diploma	76	31.9%
High school diploma	89	37.4%
College degree	19	8.0%
Occupation (n, %)		
Housekeeper	7	2.9%
Employed	17	7.1%
Unemployed	5	2.1%
Retired	209	87.8%
Smoking		
No	86	36.6%
Yes	27	11.3%
Past	122	51.3%
BMI (M, SD)	34.334	7.358
BMI class (n, %)		
Underweight (<18.5 kg/m^2^)	1	0.4%
Normal weight (18.5–24.9 kg/m^2^)	33	13.9%
Overweight (25–29.9 kg/m m^2^)	31	13.0%
Obesity class I (30–34.9 kg/m^2^)	44	18.5%
Obesity class II (35–39.9 kg/m^2^)	70	29.4%
Obesity class III (<40 kg/m^2^)	52	21.8%
HADS_Anxiety (M, SD)	5.98	4.04%
HADS_Depression (M, SD)	5.18	3.69%
HADS_Anxiety		
Mild	42	17.6%
Moderate	26	10.9%
Severe	8	3.4%
HADS_Depression		
Mild	43	18.1%
Moderate	13	5.5%
Severe	5	2.1%

M = mean; SD = standard deviation; n = number of individuals; % = percentage; BMI = body mass index.

**Table 2:** Correlations between potential determinants and outcome measures.

	QLI_Total	QLI_Health	QLI_Social	QLI_Psy	QLI_Family
BIS_AI	−0.127	−0.117	−0.138 *	−0.118	−0.055
BIS_MI	−0.059	−0.064	−0.100	−0.066	−0.006
BIS_NpI	−0.167 *	−0.165 *	−0.183 **	−0.178 **	−0.109
BIS_Total	−0.162 *	−0.159 *	−0.197 **	−0.166 *	−0.084
HADS_A	−0.430 **	−0.397 **	−0.430 **	−0.399 **	−0.279 **
HADS_D	−0.565 **	−0.538 **	−0.414 **	−0.559 **	−0.412 **
BMI	−0.344 **	−0.374 **	−0.295 **	−0.257 **	−0.235 **

** The correlation is significant at the 0.01 level (2-tailed). * The correlation is significant at the 0.05 level (2-tailed). QLI = Quality of Life Index; BIS = Barratt Impulsiveness Scale; HADS = Hospital Anxiety and Depression Scale; Health = health and functioning; Social = social and economic aspects; Psy = psychological and spiritual status; Family = family and relationships; HADS_A = anxiety; HADS_D = depression; AI = attentional impulsiveness; MI = motor impulsiveness; NpI = no-planning impulsiveness; BMI = body mass index.

**Table 3:** Hierarchical regression analysis of factors associated with quality of life.

	β	Standardized β	t	SE	*p*-Value	CI	Collinearity Diagnostics		Model Statistics
							Tolerance	VIF	
MODEL 1									R^2^ = 0.118; F(1,231) = 30.905, *p* = 0.000
BMI	−0.209	−0.344	−5.559	0.038	0.000	−0.284–−0.135	1.000	1.000	
MODEL 2									R^2^ = 0.138; F(2,230) = 18.441, *p* = 0.021
BMI	−0.205	−0.335	−5.470	0.037	0.000	−0.278–−0.131	0.997	1.003	
BIS_Total	−0.060	−0.142	−2.322	0.026	0.021	−0.111–−0.009	0.997	1.003	
MODEL 3									R^2^ = 0.276; F(4,228) = 34.315, *p* = 0.000
BMI	−0.138	−0.226	−4.214	0.033	0.000	−0.203–−0.073	0.949	1.054	
BIS_Total	−0.001	−0.002	−0.045	0.023	0.964	−0.047–0.045	0.901	1.109	
HADS_A	−0.127	−0.113	−1.637	0.078	0.103	−0.281–0.026	0.573	1.745	
HADS_D	−0.543	−0.441	−6.393	0.085	0.000	−0.710–−0.376			

β = Regression coefficient; CI = 95% confidence intervals; BMI = body mass index; BIS_Total = Barratt Impulsiveness Scale—total; HADS_A = Hospital Anxiety and Depression Scale—anxiety; HADS_D = depression.

## Data Availability

The data set for this study is available on request to interested researchers.
